# Clinical Effect of Modified Ultrasound-Guided Subclavian Vein Puncture

**DOI:** 10.1155/2023/5534451

**Published:** 2023-07-06

**Authors:** Yun-Shui Zhang, Shuang-Long Zhang, Wen-Min Guo, Tao Liu, Yu-Jie Ma

**Affiliations:** ^1^Department of Critical Care Medicine, The Air Force Characteristic Medical Center, Air Force Medical University, Beijing 100142, China; ^2^Department of Critical Care Medicine, Peking University International Hospital, Life Park Road No. 1, Life Science Park of Zhong Guancun, ChangPing District, Beijing 102206, China

## Abstract

**Objective:**

This study compared the effect of ultrasound-guided subclavian vein puncture with traditional blind puncture and the double-screen control method by evaluating the one-time puncture success and total success rates, the completion time for puncture and catheterization, and short-term complications.

**Methods:**

From January 2020 to January 2021, 72 patients with right subclavian venipuncture catheterization were collected, 12 of whom were excluded (including 3 cases of pneumothorax, 2 cases of hemothorax, 1 case of difficult positioning of thoracic deformity, 1 case of severe drug eruption, 3 cases of clavicle fracture, and 1 case of severe coagulation dysfunction). The remaining 60 cases were randomly divided into the traditional group (*n* = 30) and the improved group (*n* = 30). We record two sets of ultrasound localization time, puncture time, one-time puncture power, total puncture success rate, and short-term (24-hour) complications.

**Results:**

Compared with the traditional group, the ultrasound positioning time and puncture time in the improved group were significantly reduced and the puncture success rate was higher. There were no complications, such as incorrect arterial puncture and the occurrence of pneumothorax, in either group.

**Conclusion:**

The improved ultrasound-guided subclavian vein catheterization technique can greatly reduce the catheterization time and improve the success rate of puncture and catheterization. It can also reduce the occurrence of complications and damage to adjacent tissues. The operation is simple, fast, and easy to master, and it has a high popularization clinical value.

## 1. Introduction

Central venous catheterization is an important channel for the treatment of critically ill adult central [1–3]. The subclavian vein is not easily collapsed due to its fixed position, and the high comfort after catheterization and the low postoperative infection rate make treatment via the subclavian vein common in clinical practice. However, because the subclavian vein is adjacent to the subclavian artery and pleura, there is a high risk of pneumothorax, hematoma, arterial injury, and other complications during puncture [4–6].

It has been reported that ultrasound-guided subclavian vein puncture can reduce both complications and puncture duration and improve the puncture success rate [7, 8]. However, most previous studies do not elaborate on the specific operation methods. In the current published literature, puncture methods are divided roughly into the following three techniques: the long-axis in-plane approach, the short-axis out-of-plane approach, and ultrasound-guided axillary vein puncture into the subclavian vein. Some studies have shown that the axillary vein is greatly affected by breathing, and it has even collapsed completely in the inspiratory stage [9]. When a catheter is positioned, it often passes through the pectoralis minor muscle, leading to arm injuries [10], which are obviously uncomfortable. It has also been reported that the short-axis method of ultrasound-guided subclavian vein catheterization is better than the long axis method; however, the former cannot display the puncture track in real time, and the risks of inadvertent arterial puncture and pneumothorax are increased significantly. Therefore, the main method used is the traditional technique of ultrasound-guided subclavian vein puncture (i.e., the long-axis plane puncture method) in which the ultrasonic probe (which is not dependent on the thickness of the tube wall) is partially located on the clavicle. However, pulse and probe pressure can flatten it, the artery or vein cannot be identified quickly, and the relationship between the tissues around the blood vessels is not apparent. Consequently, the author innovatively proposed that, in clinical application, the short-/long-axis switching double-screen control method of ultrasound-guided puncture is used as it significantly improved puncture efficiency and reduced the occurrence of complications and damage to adjacent tissues.

## 2. Materials and Methods

### 2.1. General Information

**Figure 1:**
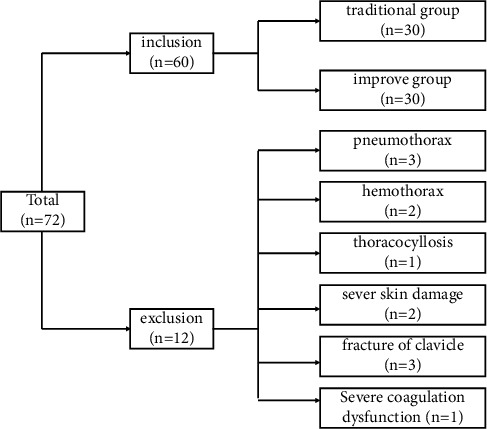
Flowchart of patient inclusion.

With the approval of the Air Force Specialty Medical Center Ethics Committee, 60 patients with right subclavian vein catheterization, which included 49 males and 11 females, were divided randomly into a traditional group (*n* = 30) and an improved group (*n* = 30).  Figure 1 showed the flow chart of patient inclusion.

### 2.2. Methods of Puncture and Catheterization

All patients were punctured under ultrasonic guidance using an Edge™ ultrasound diagnostic system (Sonosite, U.S.A.) with a 5–10 MHz high-frequency ultrasound probe. The probe was coated with a coupling agent (Zihui Technology Group Co., Ltd.) and wrapped with a sterile plastic instrument set (Jiangxi 3L Medical Products Group [Cambodia] Co., Ltd.). The depth of the probe was 3–5 cm.

All operations were performed by an attending physician with more than 5 years' experience of working in our department. All patients were placed in the supine position, with both arms on both sides of the chest wall and the face inclined to the left.

### 2.3. Control Group

The patient lay on their back, with their upper limbs hanging down on both sides of the body. Slightly abducted the right upper lamb and turned the head to the opposite side, and the neck, chest and right upper arm was completely exposed. A distance of 1 cm below the middle point of the right clavicle was taken as the puncture entry point, and the needle was inserted at an angle of 15°–20°, with the needle body close to the lower edge of the clavicle. The needle was pumped while entering, the guide wire was passed through the puncture needle, and the catheter was sent to the preinserted vein along the guide wire. After establishing the length, the guide wire was withdrawn slowly to ensure the correct positioning of the catheter tip. After successful catheterization, the skin was sutured to fix the catheter in place. The catheterization site was covered with a sterile dressing, which was changed twice a week, and the catheter was regularly checked or replaced.

### 2.4. Short-/Long-Axis Switching Double-Screen Control Method

**Figure 2:**
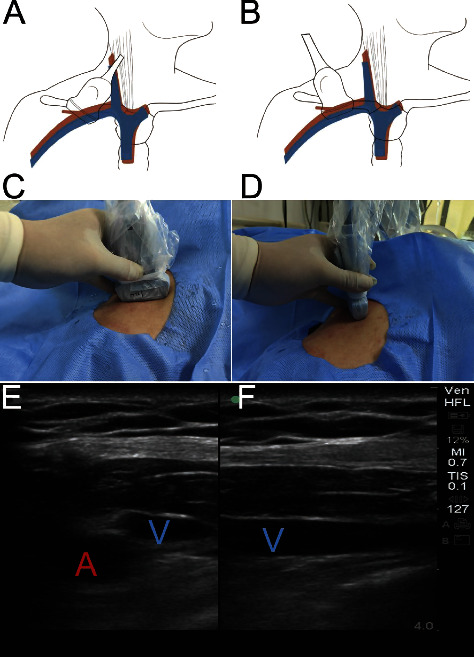
The short and long axis double screens were used to control the puncture plane. (A to C) the diagram, operating photo and ultrasound image of short axis plane. (D to E) the diagram, operating photo and ultrasound image of long axis plane. “The short and long axis double screens were used to control the puncture plane.” The value is in bold format.

The ultrasound probe was placed parallel to the clavicle by the middle point of the clavicle (the ultrasonic mark point was located at the upper right), and an appropriate section was identified to enable the subclavian artery and vein to be displayed in the center of the screen simultaneously. On the short-axis plane, according to the position relationship, fluctuations, and changes in pressure, the artery and vein could be quickly determined. The double-screen mode was selected, and the standard short-axis anatomical plane was selected on the left screen. Generally, the artery is located on the lower side, while the vein is located on the upper side. The slightly overlapped update key was selected to switch to the right screen, and the probe position was maintained as it was rotated counterclockwise (Figure 2). The subclavian vein could be quickly identified according to the tissue structure and relationship depth around the left platen vein. The right platen clearly displayed the relationship between the clavicle and the subclavian vein, and the plane method was used.

**Figure 3:**
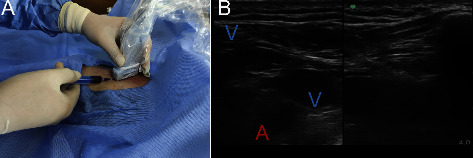
Image and photo of puncturing (A) the real‐time ultrasound image of double screen puncturing. Yellow line shows the needle trajectory. (B) The operating photo of puncturing. “Image and photo of puncturing” the value is in bold format.

In the plane, the angle of entry was 45°. When the puncture needle entered the vein, dark black blood was drawn back under negative pressure and the guide wire was inserted (Figure 3). The ultrasound confirmed that the guide wire was located in the subclavian vein, and the blood vessels in the neck were checked. If the guide wire was inserted incorrectly into the internal jugular vein, a part of the guide wire was withdrawn from that vein and reinserted until it was successfully inserted into the superior vena cava.

The structure of the subclavian vein was displayed on the left screen. At the same time, the right screen used in-plane puncture technology, which only displayed the target blood vessel, clavicle, and pleura in the plane. The target blood vessel could be punctured directly in real time for the far position of the pleura, and the clavicle needle could be inserted at a shallow angle for the near position of the pleura. Then, the needle was withdrawn by 0.5–1.0 cm, and the tail of the needle was raised close to the clavicle to complete the puncture.

### 2.5. Observation Indicators

The following indicators were observed: (1) the number of successful puncture cases after 1, 2, and 3 punctures, (2) the time from the initial puncture to the successful insertion of the guide wire, (3) the number of puncture times (when the needle tip retreated or changed the direction of entry, the needle was recorded as one time), (4) the one-time puncture success rate, (5) the total puncture success rate, and (6) complications (e.g., incorrect arterial entry, hematoma, bleeding, and nerve injury).

### 2.6. Statistical Methods

The study used SPSS 20.0 statistical software for its analysis. The chi-squared test or Fisher's exact test was used to compare count data between the groups, and the measurement data were expressed as the mean ± standard deviation, which was consistent with a normal distribution. An independent-samples *t* test was used for comparisons between the two groups. All tests were bilateral, and a value of *P*  <  0.05 was considered statistically significant.

## 3. Results

### 3.1. Differences in Puncture Success Rates

A comparison of the baseline characteristics between the patients in the experimental and control groups is shown in Table 1. Compared with each other, the one-time puncture success rate of the short-/long-axis switching group was 80%, which was better than in the short-axis plane group (66.7%); the total puncture success rate of both groups was 100%, and there was no statistical difference. In addition, the number of needle changes in the short-/long-axis switching group was less than that in the short-axis plane group, and the number of punctures in the short-/long-axis switching group was also less than that in the short-axis plane group (*P*  <  0.001) (Table 2).

### 3.2. Incidence of Complications

In the short-/long-axis switching plane method, there was one case of pneumothorax and two cases of hematoncus of the subclavian vein puncture. The incidence of complications was 10%. In the short-axis plane method, there were five cases of pneumothorax and six cases of hematoncus of the subclavian vein puncture. The incidence of complications was 37%. The incidence of complications in the short-/long-axis switching group was lower than in the short-axis group (*P*  <  0.05) (Table 3).

### 3.3. Differences in Puncture Time

The positioning time in the short-axis method was shorter than that in the control group (*P*  <  0.05). The average time for puncturing the target vessel was about 6.3333 min in the short-/long-axis switching method group and 11.3667 min in the short-axis group. The total catheterization time was shorter in the short-/long-axis switching group than in the short-axis group (*P*  <  0.05) (Table 4).

## 4. Discussion

The commonly used puncture routes included the internal jugular vein, subclavian vein, and femoral vein. The internal jugular vein is close to the oropharynx and respiratory tract and has a high rate of catheter infection and thrombosis, which limits its clinical application. The infection rate of subclavian vein catheter is low, which is convenient for nursing, but the incidence of puncture complications is high, which is easy to cause pneumothorax, and accidental artery puncture cannot stop bleeding, so the technical requirements for the operator are higher [11]. Femoral vein puncture and catheterization will not cause serious life-threatening complications. Its disadvantage is that the puncture point is in the perineum, which is easy to be infected and inconvenient for nursing.

The subclavian vein is the most commonly used puncture site for long-term catheterization in the past [12]. Although the incidence of infection and thrombosis of the subclavian vein central venous catheter is lower than that of the internal jugular vein catheter, the subclavian vein is blocked by the bone echo image of the clavicle, which affects the application of ultrasound visualization. Therefore, our innovative use of ultrasound double screen guidance for subclavian venipuncture has achieved good clinical results. With the promotion of bedside ultrasound, the use of ultrasound-guided deep vein puncture is increasing. However, most studies have focused on the puncture guidance of the internal jugular and femoral veins. Because the clavicle blocks ultrasound beams, ultrasound is not widely used in subclavian vein puncture.

Axillary vein catheterization is guided by real-time ultrasound. Studies have reported that axillary vein catheterization often passes through the pectoralis minor muscle, resulting in obvious traction discomfort when patients move their arms [11]. The risk of thrombosis in axillary vein catheterization is higher. For elderly patients, due to low subcutaneous fat accumulation, the axillary vein is thinner and easier to collapse than in the general patient population [13]. If there is also an insufficient effective circulating blood volume, the axillary vein can be completely closed when breathing, which may cause complications and make it very difficult to puncture.

The diameter of the subclavian vein is larger than that of the axillary vein, the position of the subclavian vein is fixed, and it is less affected by respiration. In the past, ultrasound-guided subclavian vein puncture was concentrated mainly in the short- and long-axis planes [14, 15]. However, the short-axis method cannot display the puncture track in real time, and the risk of puncturing artery and creating a pneumothorax is significantly increased [16–18].

In the long-axis plane puncture method, the ultrasonic probe is located partially on the clavicle, which relies on wall thickness. However, pulsation and pressure can flatten it, the artery and vein cannot be determined quickly, and the relationship between the tissue surrounding the vessels cannot be ascertained. Consequently, the author innovatively proposed that, in clinical practice, the short-/long-axis switching double-screen control method of ultrasound-guided puncture is used, which significantly improves puncture efficiency and reduces the occurrence of complications and damage to adjacent tissues.

In summary, the puncture process operation is safer, the operating technology is simple and intuitive, and the ultrasound dual-screen display function is found in the basic configuration of an ultrasound machine, which is conducive to the method's use by grass-roots hospitals. Consequently, it is a worthy technique for conducting ultrasound-guided puncture.

## Figures and Tables

**Table 1:** Patients' general information.

	Experimental group	Control group	*P*
*Gender*			0.181
Female	3	8	
Male	27	22	
*Age (years)*			0.500
<60	4	5	
≥60	26	25	
*BMI*			0.604
≥24	15	18	
<24	15	12	
*Coagulation state*			0.313
Normal	29	30	
Abnormal	1	0	

**Table 2:** The difference of the puncture success rate.

	Experimental group	Control group	*P*
*One-time success rate of puncture*			0.243
Yes	24	20	
No	6	10	
*Total puncture success rate*			1
100%	30	30	
<100%	0	0	
*Number of punctures*			<0.001
1	24	20	
2	5	5	
≥3	1	5	

**Table 3:** Complications' rate.

	Experimental group	Control group	*P*
*Pneumothorax*			0.047
Yes	1	5	
No	29	25	
*Hematoncus*			0.045
Yes	2	6	
No	28	24	

**Table 4:** Total intubation time.

	Cases	Average Time (min)	95% CI		*t*	*P*
			Lower	Upper		
Experimental group	30	6.3333	−5.731	−3.734	−9.695	<0.001
Control group	30	11.3667				

## Data Availability

The data that support the findings of this study are available from the corresponding author upon reasonable request.
